# Vitamin D Supplementation Rescues Aberrant NF-κB Pathway Activation and Partially Ameliorates Rett Syndrome Phenotypes in *Mecp2* Mutant Mice

**DOI:** 10.1523/ENEURO.0167-20.2020

**Published:** 2020-05-22

**Authors:** Mayara C. Ribeiro, Seth M. Moore, Noriyuki Kishi, Jeffrey D. Macklis, Jessica L. MacDonald

**Affiliations:** 1 Department of Biology, Program in Neuroscience, Syracuse University, Syracuse, NY 13244; 2 Department of Stem Cell and Regenerative Biology, and Center for Brain Science, Harvard University, Cambridge, MA 02138

**Keywords:** epigenetics, neocortex, neuronal morphology, NF-κB, Rett syndrome, vitamin D

## Abstract

Rett syndrome (RTT) is a severe, progressive X-linked neurodevelopmental disorder caused by mutations in the transcriptional regulator *MECP2*. We previously identified aberrant NF-κB pathway upregulation in brains of *Mecp2*-null mice and demonstrated that genetically attenuating NF-κB rescues some characteristic neuronal RTT phenotypes. These results raised the intriguing question of whether NF-κB pathway inhibitors might provide a therapeutic avenue in RTT. Here, we investigate whether the known NF-κB pathway inhibitor vitamin D ameliorates neuronal phenotypes in *Mecp2*-mutant mice. Vitamin D deficiency is prevalent among RTT patients, and we find that *Mecp2*-null mice similarly have significantly reduced 25(OH)D serum levels compared with wild-type littermates. We identify that vitamin D rescues aberrant NF-κB pathway activation and reduced neurite outgrowth of *Mecp2* knock-down cortical neurons *in vitro*. Further, dietary supplementation with vitamin D in early symptomatic male *Mecp2* hemizygous null and female *Mecp2* heterozygous mice ameliorates reduced neocortical dendritic morphology and soma size phenotypes and modestly improves reduced lifespan of *Mecp2*-nulls. These results elucidate fundamental neurobiology of RTT and provide foundation that NF-κB pathway inhibition might be a therapeutic target for RTT.

## Significance Statement

There is currently no effective treatment for Rett syndrome (RTT); however, selectively re-expressing *Mecp2* in adult mice has shown that RTT symptoms can be partially reversed, suggesting that restoration of homeostasis of downstream targets of MeCP2 could also reverse or alleviate RTT symptoms. One such potential target is the NF-κB pathway, which is aberrantly upregulated in the brain of *Mecp2*-mutant mice. Genetically reducing NF-κB signaling in these mice improves neuronal phenotypes. Here, we identify that the known NF-κB inhibitor vitamin D reduces the aberrant NF-κB signaling in *Mecp2* knock-down neurons and partially ameliorates neuronal size and complexity phenotypes in both male and female *Mecp2*-mutant mice. Thus, this simple, cost-effective dietary supplement could contribute toward a partial therapeutic avenue in RTT.

## Introduction

There is currently no effective treatment for Rett syndrome (RTT), a severe X-linked progressive neurodevelopmental disorder caused by mutations in the transcriptional regulator *MECP2* (Amir et al., 1999). Girls with this devastating disorder develop relatively normally for 6–18 months, after which they undergo a period of rapid regression, with loss of motor skills, including purposeful hand movement, deceleration of head growth, and onset of repetitive, autistic behaviors (Chahrour and Zoghbi, 2007). Importantly, selectively re-expressing *Mecp2* in adult mice has shown that RTT symptoms can be partially reversed (Luikenhuis et al., 2004; Giacometti et al., 2007; Guy et al., 2007), indicating that MeCP2 is necessary for both the development and maintenance of mature neurons (McGraw et al., 2011; Nguyen et al., 2012). These results suggest the potential for postsymptomatic therapeutic intervention, and open up the exciting prospect to at least partially stall or reverse phenotypic progression by restoring homeostasis of downstream targets of MeCP2.

One such potential downstream target is the NF-κB pathway. The NF-κB pathway regulates many cellular processes, including neural process development, structural plasticity, and learning and memory (Gutierrez and Davies, 2011). Mutations in components of the NF-κB pathway cause a spectrum of cognitive phenotypes in humans, including intellectual disability and autism spectrum disorders (ASDs; Mochida et al., 2009; Philippe et al., 2009; Manzini et al., 2014). Previously, we identified aberrant upregulation of *Irak1*, encoding a signaling kinase and scaffold protein within the NF-κB pathway, in purified cortical callosal projection neurons (CPNs) from male *Mecp2*-null mice (Kishi et al., 2016). Upregulation of *Irak1* has also been observed in different regions of the brain across RTT mouse models, correlating with phenotype severity (Gabel et al., 2015), further supporting our results. We found that *Irak1* overexpression recapitulates the reduced dendritic complexity phenotype of *Mecp2*-null CPN, and that NF-κB pathway signaling is aberrantly upregulated in cortical neurons with *Mecp2* loss-of-function. We genetically attenuated the aberrant NF-κB signaling in *Mecp2*-null mice by crossing them with mice heterozygous for *Nfkb1*. Strikingly, this genetic attenuation partially rescues the reduced cortical dendritic complexity in *Mecp2*-null mice, a hallmark of RTT that is recapitulated in these animals, and it substantially extends their normally shortened lifespan.

There are many known inhibitors of the NF-κB pathway. The known ability of vitamin D to inhibit NF-κB signaling (Chen et al., 2013b; Lundqvist et al., 2014) is particularly compelling given the high prevalence of vitamin D deficiency in RTT patients (Motil et al., 2011; Sarajlija et al., 2013). Developmental vitamin D deficiency leads to severe neurodevelopmental disruptions and behavioral abnormalities in rodents (Eyles et al., 2013; Cui et al., 2017), and there is growing evidence of a correlation between vitamin D deficiency and neurodevelopmental disorders, including ASD (Cannell, 2013; Patrick and Ames, 2014; Fernell et al., 2015), epilepsy (Hollo et al., 2014), and cognitive function (Mayne and Burne, 2019). Vitamin D supplements can improve behavioral measures in some children with ASD (Jia et al., 2015) and phenotypes in rodent models of ASD-like characteristics (Du et al., 2017; Vuillermot et al., 2017). The precise mechanisms by which vitamin D regulates neurodevelopment are not known, and might include modulation of NF-κB as well as parallel pathways.

Here, we investigated whether vitamin D supplementation can inhibit the aberrant NF-κB signaling in cortical neurons that occurs with *Mecp2* loss-of-function and whether such supplementation can ameliorate RTT phenotypes in male and female *Mecp2* mutant mice. We determined that addition of the activated form of vitamin D rescues the increased NF-κB-dependent transcription that occurs with *Mecp2* knock-down and increases neurite outgrowth *in vitro*. Further, we employed custom chow to discover that dietary vitamin D supplementation *in vivo* rescues the neuronal morphology of both male *Mecp2*-null and female heterozygous mice, and modestly extends the lifespan of male *Mecp2*-nulls. These results provide proof-of-concept that NF-κB pathway inhibition, including via vitamin D supplementation, could provide a novel therapeutic target for some RTT phenotypes.

## Materials and Methods

### Experimental design and statistical analyses

Animals were placed on custom chow in a rotating order based on date of birth. In rare instances when a litter contained three or more nulls or heterozygous females, the mice were randomly divided into two cages by an investigator blinded to experimental conditions, and were treated as sequential litters to avoid over-representation of littermates in one treatment group. Mice were weighed weekly, and assessed with a phenotypic score following criteria established by Guy et al. (2007) by an investigator blinded to genotype and chow concentration. In brief, the mice were evaluated for abnormal gait, hindlimb clasping, irregular breathing, tremor, impaired mobility and poor general body condition. Each symptom was scored as 0 (absent), 1 (present), or 2 (severe), and the score for each symptom was summed to provide an overall phenotype score with a maximum possible score of 12. Any mouse scoring a 2 (highly symptomatic) for general body condition, tremor, or breathing, or that lost >20% of presymptomatic body weight was euthanized, and the day of euthanasia was considered day of death for lifespan analysis. The selection of sample size was based on standards in the field, and on criteria established by the RTT research community (Katz et al., 2012). All morphologic and phenotypic analyses were performed by investigators blinded to experimental conditions (genotype and treatment group).

GraphPad Prism 8.0 (GraphPad Software) was used to carry out the statistical analyses. No statistical methods were used to predetermine sample sizes, but our sample sizes are similar to those generally employed in the field. Our statistical tests consisted of two-tailed *t* test, one-way ANOVA with Tukey’s multiple comparison, or two-way ANOVA with Bonferroni *post hoc* test analyses to determine statistical significance between groups. Data distribution was handled as if normal, but this was not formally tested (since potential differences in results would be minor). Variance between groups was analyzed using the *f* test procedure. For the survival curve analysis, we used the log-rank test, since this method is commonly used to compare the survival distributions of two groups. All data shown represent mean ± SEM. Sample size and statistical test are specified in each figure legends.

### Animals

All animal experimental protocols were approved by the Harvard University and/or Syracuse University Institutional Animal Care and Use Committee and adhere to NIH guidelines. Mice were group housed at a maximum of five mice per cage on a 12/12 h light/dark cycle and were given food and water *ad libitum*. CD-1 timed pregnant female mice were purchased from Charles River. Female *Mecp2* heterozygous mice were purchased from The Jackson Laboratory (B6.129P2(C)-*Mecp2*^tm1.1Bird^/J; RRID:IMSR_JAX:003890), and were maintained on a C57BL/6 background. Genotypes were determined by PCR on genomic DNA as follows: *Mecp2* mutant mice, forward primer oIMR1436 5′- GGT AAA GAC CCA TGT GAC CC −3’; reverse primer oIMR1437 5′- TCC ACC TAG CCT GCC TGT AC −3’; reverse primer oIMR1438 5′- GGC TTG CCA CAT GAC AA-3′.

### Constructs

To knock down *Mecp2* expression, a construct consisting of a bicistronic cassette encoding an shRNA sequence targeted against *Mecp2* driven by a U6 promoter, and GFP driven by a ubiquitin promoter, was used. In control experiments, a scrambled sequence replaced the *Mecp2* shRNA (both constructs were a generous gift of Z. Zhou, University of Pennsylvania; Zhou et al., 2006). To measure NF-κB activation, a plasmid containing 5 copies of an NF-κB response element (NF-κB-RE) driving expression of the luciferase reporter gene luc2P was purchased from Promega (catalog #E8491). Relative luminescence was normalized to a co-transfected *Renilla* luciferase construct, derived from the psiCHECK-2 vector (Promega, catalog #C8021) with the HSV-TK promoter and Firefly luciferase cut out by digestion with NotI and XbaI.

### Embryonic cortical neuron culture

Embryonic day (E)15.5 embryos were collected from timed pregnant CD-1 mice and the cortex was dissected out in dissociation medium (DM) containing MgKyn (Sigma-Aldrich), glucose, AP-V (Sigma-Aldrich), penicillin-streptomycin (Invitrogen), and B27 supplement (Invitrogen). The cells were dissociated using cysteine (Sigma-Aldrich), papain, and OptiMem media. Glass coverslips were precoated with poly-D-lysine hydrobromide (Sigma-Aldrich P-6407). For neurite outgrowth experiments, 5 million cells were electroporated with 20 μg of either *shScram* or *shMecp2* plasmid (BTX ECM 830 Square Wave Electroporation system, following the parameters: 700 V, one unipolar pulse at 100-μs pulse length in a 100-ms interval). After a recovery period of 5 min, 50,000 cells per coverslip were plated in neurobasal based medium containing Glutamax (Invitrogen), fetal bovine serum (Invitrogen), and penicillin-streptomycin (Invitrogen). After 4 h, the plating medium was removed, and growth medium was added, which contained Neurobasal (Invitrogen), Glutamax (Invitrogen), penicillin-streptomycin (Invitrogen), N2 and B27 supplements (Invitrogen). Calcitriol treatment started on day *in vitro* (DIV)2 and continued until the cells were fixed on DIV7. For p65 nuclear quantification experiments, 50,000 cells were plated per coverslip immersed in plating medium for 4 h before being replaced by growth media. On DIV3, cells were transfected via lipofectamine 2000 (Invitrogen) with 1 μg/μl of either *shScram* or *shMecp2* plasmid, following manufacturer guidelines. Calcitriol treatment started on DIV4 and lasted until the cells were fixed on DIV14; 10 μm calcitriol stock solution was prepared by dissolving 1α,25-dihydroxyvitamin D_3_ (Sigma-Aldrich) in ethanol. For the no treatment group, only growth medium was added; for the vehicle group, only ethanol was added; for the treatment group, 100 nm of calcitriol stock solution was added. The final ethanol concentration for both the vehicle and calcitriol groups was 1%. Growth medium was changed every other day.

### Immunocytochemistry

Coverslips containing DIV7 or DIV14 cells were fixed with 4% paraformaldehyde in PBS for 15 min, followed by three PBS washes. The cells were blocked with 8% goat serum, 10% Triton X-100, and 0.3% bovine serum albumin (Sigma-Aldrich) in PBS for 20 min. The coverslips were then incubated in primary antibodies diluted in blocking solution for 1 h. Coverslips were rinsed three times with PBS for 5 min each and incubated in secondary antibodies diluted in blocking solution for 1 h. The coverslips were washed three times with PBS, rinsed with 1/3 PB, and mounted on a slide in Fluoromount (SouthernBiotech) before imaging. Antibody dilutions were as follows: rabbit α-MeCP2 (1:500, Cell Signaling Technology catalog #3456, RRID:AB_2143849), chicken α-GFP IgY (1:1000, Thermo Fisher Scientific catalog #A10262, RRID:AB_2534023), rabbit α-NF-κB P65 (1:500, Cell Signaling Technology catalog #8242, RRID:AB_10859369), and mouse α-MAP2 (1:1000, Sigma-Aldrich catalog #M1406, RRID:AB_477171). Secondary antibodies from Invitrogen Alexa Series were used based on the primary antibody dilution (1:500 or 1:1000, Invitrogen).

### p65 nuclear quantification

Cortical neurons positive for both GFP and MAP2 were imaged with a Nikon Ni-U upright fluorescence microscope with a Zyla CMOS digital camera. Three independent experiments were performed, and 6–10 neurons per condition were imaged from each experiment. DAPI was used to identify the nucleus, and p65 translocation was quantified using ImageJ as corrected total cell fluorescence [CTCF = integrated density – (area of selected cell × mean fluorescence of background readings)] (Burgess et al., 2010; McCloy et al., 2014). Images were assembled using Photoshop CC 2017 (Adobe).

### NF-κB luciferase reporter assays

Postnatal day (P)1 C57BL/6 wild-type brains were dissected and dissociated as described for embryonic cultures. Dissociated cells were nucleofected with the NF-κB reporter construct and control *Renilla* luciferase construct, along with either scrambled shRNA or *Mecp2* shRNA constructs, using an Amaxa Mouse Neuron Nucleofector kit (Lonza) and the Amaxa Nucleofector II Device (Lonza). Cells were cultured for 48 h at high density in 96-well plates coated with poly-D-lysine (Sigma-Aldrich), in growth medium composed of 50% DMEM-F12 and 50% Neurobasal (Invitrogen), with N2, B27, and GlutaMax supplements (Invitrogen). Calcitriol or vehicle control was added at 24 h. At 48 h, Firefly and *Renilla* luciferase activities were measured using the Dual-Glo Luciferase Assay system (Promega) and a GloMax 96 microplate luminometer (Promega). The luminescence of each well was normalized individually, and triplicate wells were averaged within each experiment. Relative luminescence was normalized to the control, *shScram* experimental condition, and data represent four independent biological replicates.

### Quantitative real-time PCR (qPCR)

RNA was extracted using TRIzol (Invitrogen), and cDNA was synthesized using iScript cDNA synthesis kit (Bio-Rad Laboratories) or qScript cDNA SuperMix (Quanta Biosciences). qPCR was performed on a CFX Connect Real-Time System (Bio-Rad Laboratories) according to the manufacturer’s instructions. Primer pairs for *Mecp2*, *Irak1*, *Gapdh*, and *S16* were as follows; each primer of each primer pair was designed in different exons, so as not to amplify genomic DNA:

*Irak1*: forward 5′- GCTGTGGACACCGATACCTT −3′; reverse 5′- GGTCACTCCAGCCTCTTCAG −3′;

*Gapdh*: forward 5′- GGCATTGCTCTCAATGACAA −3′; reverse 5′- TGTGAGGGAGATGCTCAGTG −3′;

*S16*: forward 5′- CACTGCAAACGGGGAAATGG −3′; reverse 5′- TGAGATGGACTGTCGGATGG −3′;

*Mecp2*: forward 5′- TATTTGATCAATCCCCAGGG −3′; reverse 5′- CTCCCTCTCCCAGTTACCGT −3′.

We used PerfeCTa SYBR Green FastMix (Quanta Biosciences) Master mix, and each PCR consisted of 1× LightCycler FastStart DNA Master SYBR Green I mixture, 0.2 μm primers, and cDNA. We used the mean of *Gapdh* and *S16* expressions as the reference gene. Each sample was run in triplicate and averaged. The relative quantification analysis was performed as follow: ΔCq = Cq of gene of interest – geometric mean of Cq of reference genes; ΔΔCq = ΔCq – mean of ΔCq of wild-type samples; fold change = 2^-ΔΔCq^. We also performed melt curve analysis to verify the specificity of the amplicons.

### Vitamin D serum measurements

Serum was collected from four pairs of *Mecp2*+/y and *Mecp2*−/y littermates at eight weeks of age, following standard protocols. Total serum 25(OH)D levels were measured by radioimmunoassay by Heartland Assays. For measurement of the vitamin D supplemented animals, three to four serum samples of *Mecp2*+/y and *Mecp2*−/y littermates at eight weeks of age and three to four serum samples of *Mecp2*+/+ and *Mecp2* +/− littermates at five months of age on the different concentrations of vitamin D were analyzed via mass spectrometry by ZRT Laboratories.

### Vitamin D supplementation

Custom chow obtained from Bio-Serv was based on the AIN-93G Rodent Diet, varying only in Vitamin D_3_ concentration. Male *Mecp2*+/y and *Mecp2*−/y littermates, and female *Mecp2*+/+ and *Mecp2*+/− littermates were each weaned together at four weeks of age, and placed on chow containing 1 IU/g vitamin D (standard chow), 10 IU/g, or 50 IU/g in rotating order based on date of birth. As per established standards for preclinical studies in *Mecp2*-null mice (Katz et al., 2012), 15–18 *Mecp2*−/y mice were analyzed for lifespan and phenotypic progression for each vitamin D concentration. Mice were weighed weekly, and assessed with a phenotypic score following criteria established by Guy et al. (2007), by an investigator blinded to genotype and chow concentration. Any mouse scoring a 2 (highly symptomatic) for general body condition, tremor, or breathing, or that lost >20% of presymptomatic body weight, was euthanized, and the day of euthanasia was considered day of death for lifespan analysis.

### Golgi staining, dendrite and soma measurements

For dendrite and soma size analyses, four to five mice of each sex and genotype were analyzed per condition, as per established standards. Mice were euthanized with avertin overdose (at eight weeks of age for males and five months of age for females), and brains were immersed in freshly prepared Golgi impregnation solution (FD Rapid GolgiStain kit; FD Neurosciences). Brains were processed according to the protocol provided by the company. Neurons were systematically selected for analysis, and imaged by an investigator blinded to genotype and experimental condition with the following *a priori* selection criteria: (1) overall cellular morphology of superficial layer cortical pyramidal neurons; (2) dendritic trees well impregnated, and not obscured by stain precipitate, blood vessels, or astrocytes; and (3) the entire dendritic tree appearing intact and visible within the 150-μm thickness of the section. Neurons were imaged on a Nikon Ni-U upright microscope with a Zyla CMOS digital camera under a 20× objective, equipped with an Optiscan XYZ motorized stage to allow for Z-stacks. NIS-Elements software was used for simple deconvolution and extended depth of focus, after which the neurons were traced using Adobe Illustrator CS5 (Adobe). Dendritic complexity was quantified using Sholl analysis (Sholl, 1953), employing ImageJ (W. S. Rasband, ImageJ, National Institutes of Health) with the Sholl Analysis Plugin (v1.0; Ghosh Lab, www.ghoshlab.org/software/index.html). The following parameters were used for dendrite analysis: step = 10 μm, beginning radius = 20 μm, final radius = 200 μm.

### Dendritic spines measurements

For apical dendritic spine density quantification, three *Mecp2*−/y and *Mecp2*+/− littermates were analyzed per condition. Neurons were selected and imaged by an investigator blinded to genotype and experimental condition, following the criteria: (1) morphology of superficial layer cortical pyramidal neurons; (2) well impregnated dendritic trees; and (3) the entire apical dendritic tree appearing intact. Neurons were imaged under a 60×-oil objective using with a Nikon Ni-U upright microscope with a Zyla CMOS digital camera, and Optiscan XYZ motorized stage, enabling Z-stacks. For the quantification, we used the software RECONSTRUCT following the directions described (Risher et al., 2014). Images were reconstructed using Photoshop CC 2017 (Adobe).

## Results

### Vitamin D serum levels are reduced in *Mecp2−*/y mice

**Figure 1.**
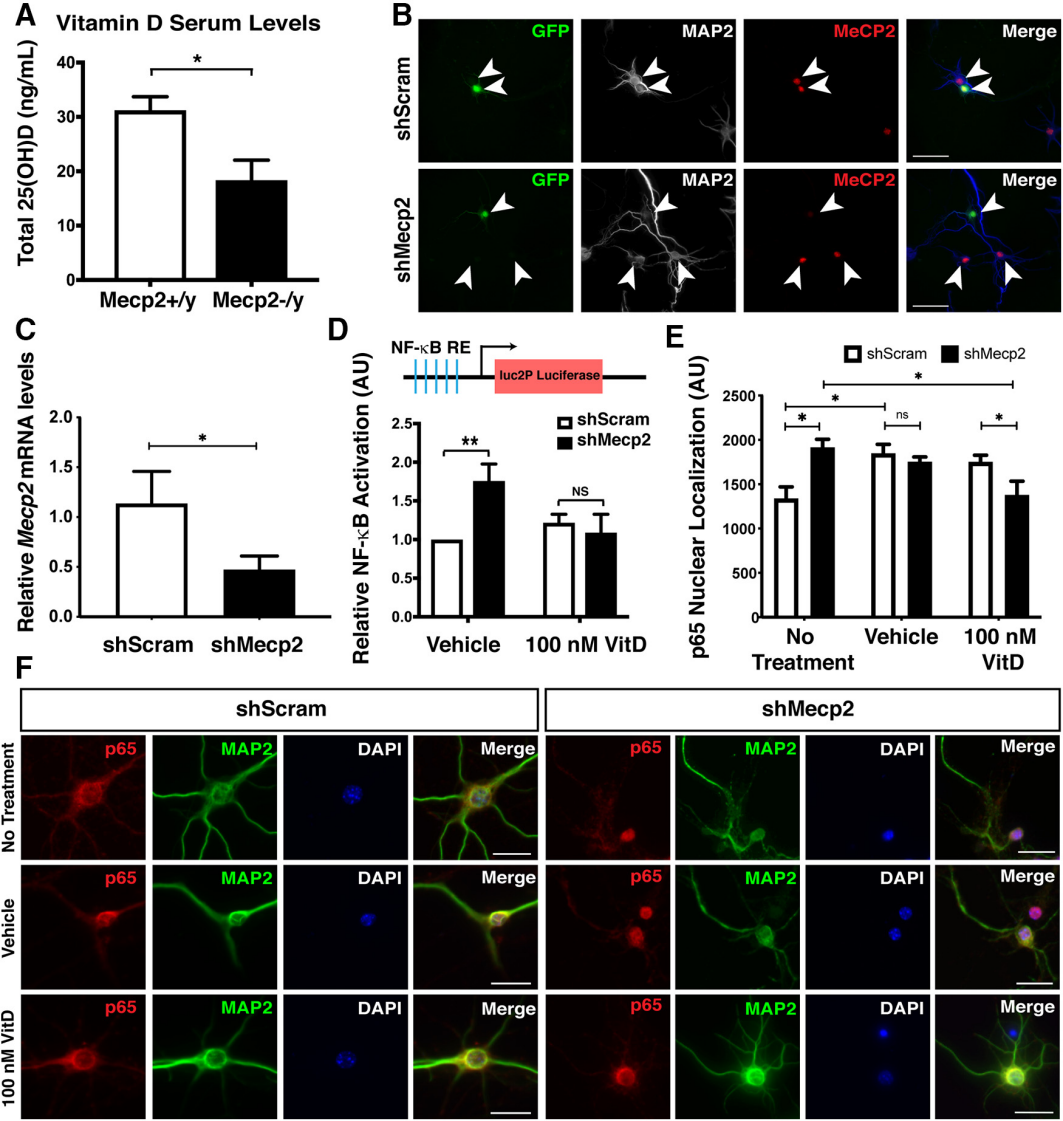
Vitamin D rescues aberrant NF-κB activation in *Mecp2* knock-down cortical neurons. ***A***, *Mecp2*-null mice have reduced serum vitamin D levels [25(OH)D] compared with the wild-type littermates at eight weeks of age (*N* = 4 mice/genotype). ***B***, ***C***, E15.5 cortical neurons were nucleofected with a construct expressing GFP as reporter and either a control shRNA (*shScram*) or an shRNA targeting *Mecp2* (*shMecp2*). *shMecp2* nucleofection visibly reduced the expression of MeCP2 protein at 7 DIV (***B***) and downregulated the overall expression of *Mecp2* ∼50% after 14 DIV, in cultures in which the transfection efficiency was ∼60% (***C***). Arrowheads indicate nucleofected GFP-positive neurons; arrows indicate neighboring non-nucleofected GFP-negative neurons. *N* = 4 experimental replicates. ***D***, Dissociated P1 cortical neurons were nucleofected with *shScram* or *shMecp2*, then were cultured for 2 d. Addition of calcitriol, the activated form of vitamin D (VitD), to culture medium for 24 h rescues the ∼1.75-fold increase in NF-κB-dependent transcription that occurs with knock-down of *Mecp2* in cortical neurons *in vitro*. However, calcitriol has no effect on *shScram* control nucleofected neurons (*N* = 4 biological replicates). ***E***, ***F***, *Mecp2* knock-down results in increased nuclear p65 localization in cortical neurons, which is indicative of NF-κB activation. Addition of calcitriol to the culture medium reduces p65 protein expression in the nucleus of *Mecp2* knock-down cortical neurons, but not control (*shScram*) neurons. ***C***, ***D***, *N* = *shScram* no treatment: 33 neurons, vehicle: 30 neurons; 100 nm VitD: 33 neurons; *shMecp2* no treatment: 23 neurons, vehicle: 22 neurons; 100 nm VitD: 22 neurons from three independent experiments. Expression of GFP was employed to identify transfected neurons. AU = relative luminescence units. ***A***, ***B***, ***D***, Two-tailed *t* test. ***E***, One-way ANOVA with Tukey’s multiple comparison test; *p < 0.05, **p < 0.01, NS = not significant. Scale bar = 20 μm. Error bar: ±SEM.

The high prevalence of vitamin D deficiency in RTT patients (Motil et al., 2011; Sarajlija et al., 2013), and the known ability of vitamin D to inhibit the NF-κB pathway (Stio et al., 2007; Chen et al., 2013b; Lundqvist et al., 2014), which is upregulated in brains of hemizygous null (*Mecp2*-/y) male mice (Kishi et al., 2016), raises the intriguing questions of whether this simple, cost-effective dietary supplement might rescue the aberrant NF-κB pathway activation in these mice, and whether it can contribute to phenotypic improvement. To investigate this and to further test the mechanistic motivation for this approach, we first analyzed vitamin D levels in the serum of eight-week-old *Mecp2*-null mice and wild-type littermates (*Mecp2*+/y) by radioimmunoassay. Previous studies employing dietary vitamin D supplementation in mice have demonstrated that 1,25(OH)_2_D_3_ levels in the brain corelate with plasma 25(OH)D_3_ levels (Spach and Hayes, 2005); thus, we measured plasma 25(OH)D_3_ levels. We found that, similar to RTT patients, *Mecp2*-null mice have significantly reduced (∼50%) total serum 25(OH)D levels compared with wild-type littermates (Fig. 1*A*), further suggesting that vitamin D supplementation might have therapeutic benefit.

### Vitamin D supplementation rescues aberrant NF-κB activation in cortical neurons *in vitro*

Vitamin D and its analogues have been found to inhibit the NF-κB pathway, but this has not been well-studied in neurons. In the inactive state, the NF-κB dimer is tethered in the cytoplasm by inhibitor of κB (IκB). When the pathway is activated, IκB is phosphorylated, targeting it for proteasomal degradation. The NF-κB dimer is thus released, and translocates to the nucleus, where it binds to consensus NF-κB-REs in the DNA to activate transcription of target genes. The predominant form of NF-κB in the nervous system is a p65/p50 heterodimer (Gutierrez and Davies, 2011), and NF-κB subunits are expressed throughout the CNS, by neurons as well as by glia.

To investigate whether vitamin D supplementation can rescue aberrant NF-κB activation resulting from *Mecp2* knock-down in cortical neurons, we first employed an *in vitro* NF-κB-RE luciferase assay. We previously employed this reporter construct with tandem NF-κB-REs and a minimal reporter driving luciferase to assay NF-κB transcriptional activity in cortical neurons following *Irak1* overexpression or *Mecp2* knock-down, and identified significant upregulation of NF-κB dependent transcriptional activity (Kishi et al., 2016). We employed an shRNA-mediated *Mecp2* knock-down approach in wild-type neurons, allowing for an efficient, higher-throughput *in vitro* system; the high transfection efficiency obtained with the nucleofection approach (∼60% of surviving cells) recapitulates the heterogeneous MeCP2 expression of a *Mecp2*+/− cortices. The *Mecp2* knock-down and control shRNA constructs employed have been previously validated and published (Zhou et al., 2006; Wood et al., 2009; Kishi et al., 2016). Both constructs contain eGFP driven by an independent promoter. This shRNA-mediated knock-down of *Mecp2* is sufficient to visibly reduce, but not eliminate, protein detection by immunocytochemistry in cortical neurons (Fig. 1*B*,*C*). In the vehicle control, *Mecp2* knock-down results in an approximate 1.75-fold increase in NF-κB-dependent transcriptional activity relative to *shScram*, which is similar to the previously published ∼2-fold increase observed with *Mecp2* knock-down without treatment (Kishi et al., 2016). We treated neurons with the bioactive form of vitamin D (1α,25-dihydroxyvitamin D_3_; calcitriol) for 24 h before performing NF-κB-RE luciferase assays. We find that addition of calcitriol has no effect on relative NF-κB activation in control neurons but significantly reduces the elevated NF-κB signaling in *Mecp2* knock-down neurons, bringing the level back down to that of control neurons (Fig. 1*D*).

Next, we investigated whether calcitriol might also reduce nuclear translocation of the p65 subunit of NF-κB, which is indicative of NF-κB pathway activation. For these experiments, E15.5 cortical cells were dissociated and cultured for 14 d. They were transfected with either *shScram* or *shMecp2* at 3 DIV and treated with either vehicle (ethanol) or 100 nm calcitriol starting at 4 DIV. Transfected neurons were identified by co-expression of GFP and the neuronal marker MAP2. *shMecp2*-transfected neurons express higher levels of p65 protein in their nucleus compared with control (*shScram*)-transfected cells (Fig. 1*E*, left). Interestingly, the addition of the vehicle (EtOH) increases p65 nuclear translocation in control transfected neurons, but not in *Mecp2* knock-down neurons, perhaps indicating that pathway activation is already maximal in these neurons (Fig. 1*E*, middle). Ethanol is known to alter NF-κB signaling via ROS-dependent pathways, increasing p65 phosphorylation and its nuclear translocation in neurons and glia (Davis and Syapin, 2004; Lippai et al., 2013; Okabe et al., 2016; Vetreno and Crews, 2018). However, addition of 100 nm calcitriol reduces p65 nuclear localization in *Mecp2* knock-down neurons without affecting the control neurons (Fig. 1*E*,*F*), indicating that vitamin D supplementation can reduce aberrant NF-κB signaling in *Mecp2*-deficient cortical neurons *in vitro*.

### Vitamin D rescues reduced neurite outgrowth of *Mecp2* knock-down cortical neurons *in vitro*

**Figure 2.**
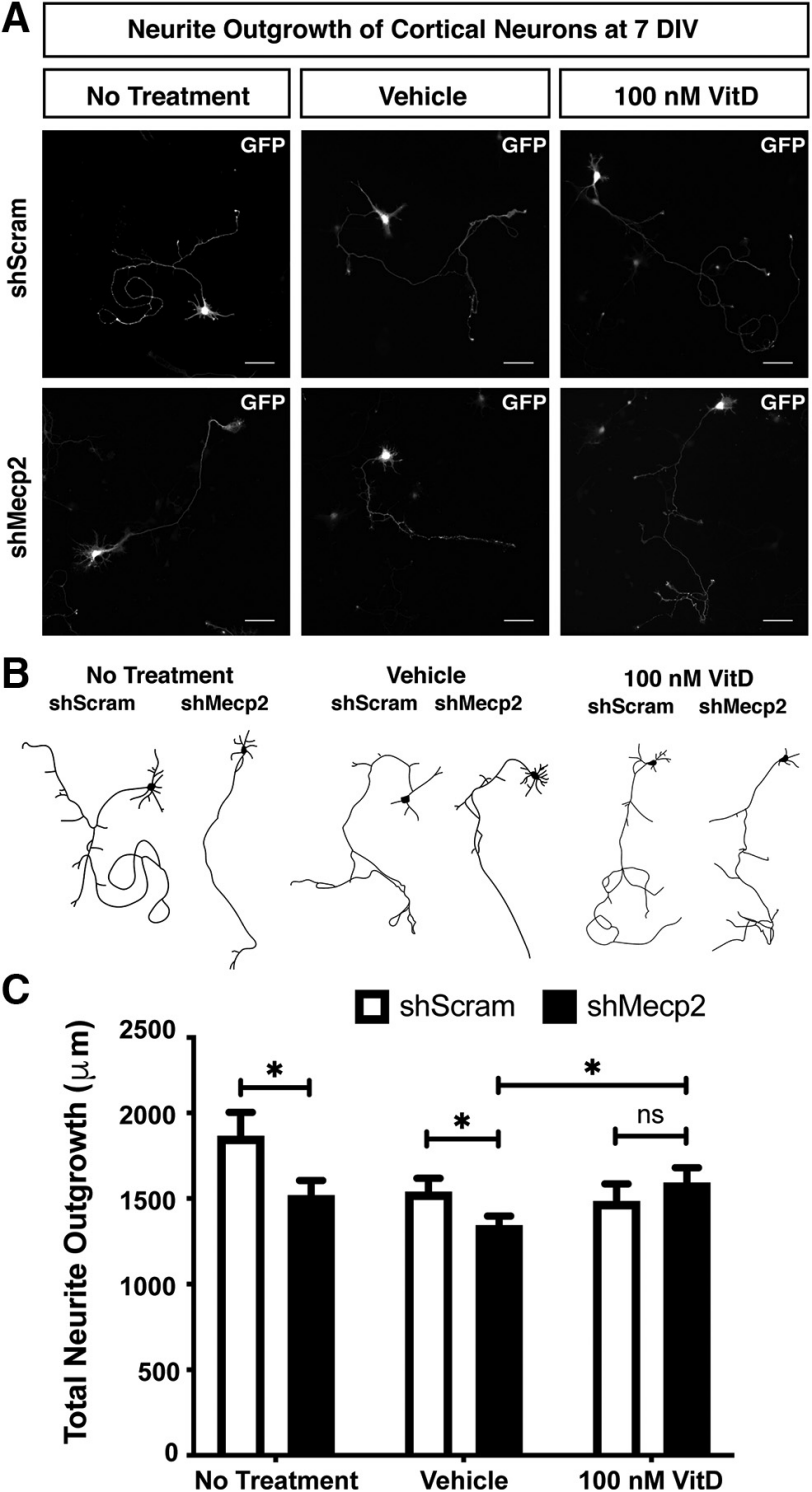
Vitamin D rescues reduced neurite outgrowth of *Mecp2* knock-down cortical neurons. ***A–C***, Dissociated E15.5 cortical neurons were nucleofected with a construct expressing a GFP reporter and either a control shRNA (*shScram*) or an shRNA targeting *Mecp2* (*shMecp2*), then were plated and cultured for 7 d. Neurons were either maintained in standard culture medium, or were supplemented with vehicle (EtOH) or 100 nm calcitriol (VitD) from 2 to 7 DIV. ***A***, Representative images of GFP+ cortical neurons at 7 DIV under each condition. ***B***, Representative traces of GFP+ cortical neurons under each condition. ***C***, Total neurite outgrowth of GFP+ neurons was quantified from randomly selected neurons, from each of three independent experiments (*N* = *shScram* no treatment: 30 neurons, vehicle: 26 neurons; 100 nm VitD: 27 neurons; *shMecp2* no treatment: 28 neurons, vehicle: 26 neurons; 100 nm VitD: 27 neurons). Supplementation with calcitriol rescues the reduced neurite outgrowth of *Mecp2* knock-down neurons relative to EtOH vehicle control but does not have a significant effect on control cortical neurons. Thus, *shMecp2* neurons with calcitriol are not significantly different from *shScram*. ***C***, One-way ANOVA with Tukey’s multiple comparison test; *p < 0.05, NS = not significant. Scale bar = 50 μm. Error bar: ±SEM.

We next investigated whether addition of calcitriol might also rescue the reduced neurite outgrowth of *Mecp2* knock-down neurons *in vitro*. E15.5 cortical neurons transfected with *shMecp2* demonstrate a significant reduction in total neurite outgrowth by 7 DIV, in comparison to control *shScram* transfected neurons (Fig. 2*A–C*). Transfected neurons were again identified by GFP and MAP2 expression, with GFP used to trace total neurites. Calcitriol or ethanol (vehicle) was added to the culture medium from 2 to 7 DIV (Fig. 2*A*,*B*). The vehicle marginally reduced the total neurite outgrowth of both *shScram* and *shMecp2* neurons; however, *Mecp2* knock-down neurons have significantly decreased neurite length compared with *shScram* control, both in untreated and vehicle-treated cultures. Ethanol has also been shown to negatively alter neuronal dendritic complexity and neurite development, as discussed above for p65 localization. However, there is no difference in total neurite outgrowth between vehicle and calcitriol treated *shScram* neurons, while there is a significant increase in total neurite outgrowth of *shMecp2* neurons treated with calcitriol compared with vehicle. (Fig. 2*C*). Together, these data indicate that vitamin D is able to act on neurons to modify NF-κB signaling and *Mecp2* knock-down cortical neuronal phenotypes *in vitro*, thus motivating investigation of how vitamin D modifies *Mecp2*-mutant neuronal phenotypes *in vivo*.

### Dietary vitamin D supplementation moderately extends the reduced lifespan of *Mecp2*-null mice

**Figure 3.**
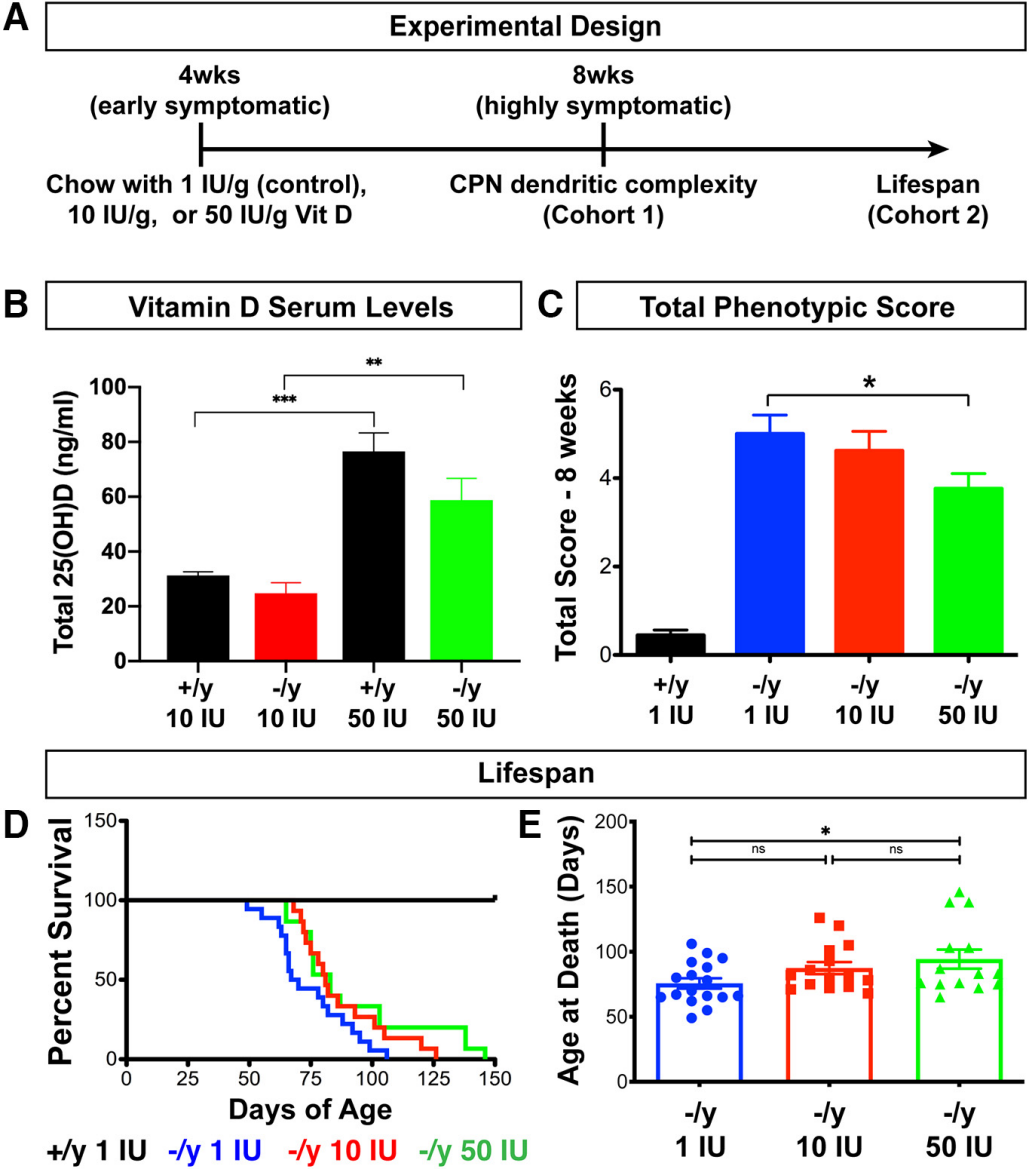
Vitamin D supplementation modestly improves *Mecp2*-null phenotypes and increases their reduced lifespan. ***A***, Experimental plan for *in vivo* vitamin D treatment of *Mecp2*−/y and *Mecp2*+/y littermates. ***B***, Supplementing the diet of the mice with vitamin D (VitD) significantly increases their total serum levels of 25(OH)D, regardless of genotype, which is most apparent with 50 IU/g supplemented chow. ***C***, *Mecp2*−/y on 50 IU/g VitD have a small, but significant, reduction in total phenotypic score at eight weeks of age compared with *Mecp2*−/y on control 1 IU/g VitD. ***D***, Kaplan–Meier survival curves. *Mecp2*−/y mice on 50 IU/g VitD chow survive significantly longer than *Mecp2*−/y mice on control chow, while *Mecp2*−/y mice on 10 IU/g VitD display a trend toward increased median lifespan (*p* = 0.04; log-rank test). The median lifespan of *Mecp2*−/y on 1 IU/g is 68.5 d, 81 d on 10 IU/g, and 83 d on 50 IU/g. ***E***, The mean age of death of *Mecp2*-/y mice on the control chow is significantly lower than the animals on 50 IU/g VitD. ***B***, ***C***, ***E***, One-way ANOVA with Tukey’s multiple comparison test; *p < 0.05, **p < 0.01, ***p < 0.001, NS = not significant. ***B***, *N* = 4 mice per condition. ***C–E***, *N* = 16 *Mecp2*+/y 1 IU, 17 *Mecp2*-/y 1 IU, 15 *Mecp2*-/y 10 IU, 14 *Mecp2*-/y 50 IU. Error bar: ±SEM.

To investigate whether vitamin D supplementation might also improve specific *Mecp2*-null phenotypes *in vivo*, we treated *Mecp2*-null (*Mecp2*^tm1.1Bird^) and wild-type littermates with vitamin D supplemented chow and analyzed complexity and soma size of cortical neurons in one cohort of mice and overall phenotypic progression and (morbidity-limited) lifespan in a second cohort (Fig. 3*A*). *Mecp2*-null and wild-type littermates were placed on chow containing one of three vitamin D concentrations in a strict rotation based on date of birth: 1 IU/g (standard chow concentration, serving as control), 10 IU/g, or 50 IU/g vitamin D. Chow with 10 IU/g and 50 IU/g are well tolerated and can alter neuronal pathology (Gianforcaro and Hamadeh, 2012; Gianforcaro et al., 2013; Latimer et al., 2014). A pilot study included chow with 200 IU/g vitamin D, which is well below the published toxic range; however, we found that it led to reduced lifespan in *Mecp2*-/y mice, and thus, this dosage was halted. Mice on a diet supplemented with 50 IU/g of vitamin D have more than a twofold increase in total 25(OH)D serum concentration compared with the mice on 10 IU/g of vitamin D, regardless of genotype (Fig. 3*B*), indicating that the dietary supplementation is effective at increasing circulating vitamin D in *Mecp2*−/y mice, beyond that observed in *Mecp2*+/y mice under control conditions (Fig. 1*A*).

At four weeks of age, *Mecp2*-/y mice are mildly symptomatic, already demonstrating reduced body weight relative to wild-type littermates, and a small, but significant increase in phenotypic score (data not shown). Dendritic complexity and soma size of Layer II/III CPN are not significantly disrupted in *Mecp2*-null mice at four weeks of age but are significantly reduced compared with wild-type by eight weeks of age (Kishi and Macklis, 2004). *Mecp2*-nulls display an overall rapid phenotypic progression between four and eight weeks of age and a median survival between 10 and 11 weeks (Guy et al., 2001); we thus treated the mice with vitamin D during this critical window.

The mice were weighed weekly and assessed with a phenotypic score (Guy et al., 2007) by an investigator blinded to genotype and chow concentration. Briefly, the mice were assigned a score of 0 (absent), 1 (present), or 2 (severe) for each of the following six phenotypes: abnormal gait, hindlimb clasping, irregular breathing, tremor, impaired mobility, and poor general body condition. The score for each symptom was summed to provide an overall phenotype score, with a maximum possible score of 12.

While vitamin D supplementation does not significantly alter the reduced weight of *Mecp2*−/y mice, *Mecp2*−/y mice on 50 IU/g vitamin D demonstrate a small, but significant, reduction in total phenotypic score by eight weeks of age (Fig. 3*C*). Thus, to investigate whether vitamin D supplementation can, indeed, slow broad phenotypic progression, we analyzed lifespan as an indicator of overall phenotypic progression and health. Following established standards for preclinical trials in *Mecp2* mutant mice (Katz et al., 2012), 14–17 *Mecp2*-null mice and wild-type littermates were maintained on each vitamin D concentration from four weeks of age until death.

Supplementation with 50 IU/g significantly increased the median lifespan of *Mecp2*-null mice (83 d, log-rank test *p* = 0.04), while supplementation with 10 IU/g vitamin D produced a trend to increased median lifespan (from 68.5 d on control chow to 81 d; Fig. 3*D*). The mean age at death is significantly increased for *Mecp2*−/y mice on 50 IU/g vitamin D, relative to those on the control chow (Fig. 3*E*). While this ∼20% increase in survival is not as extensive as that obtained with genetic attenuation of NF-κB signaling (Kishi et al., 2016), it is similar to results seen with other treatments currently under investigation, such as human recombinant IGF1 (Castro et al., 2014). Taken together, these results provide highly intriguing evidence that dietary supplementation with vitamin D might provide a partial improvement of some RTT phenotypes.

### Dietary vitamin D supplementation rescues projection neuron dendritic complexity and soma size phenotypes in *Mecp2-*/y neocortex

To investigate a specific RTT neuronal phenotype that is recapitulated in *Mecp2* mutant mice, we analyzed the complexity and soma size of Layer II/III CPN. CPN, the broad population of commissural neurons whose axons connect the two cerebral hemispheres via the corpus callosum (CC), are excitatory pyramidal projection neurons whose cell bodies reside in neocortical Layers II/III (∼80% in mouse), V (∼20%), and a few % in VI (Fame et al., 2011). Layer II/III CPN increasingly express MeCP2 as they mature, and loss of MeCP2 function reduces their dendritic complexity in a largely cell-autonomous manner (Kishi and Macklis, 2004, 2010). Reduced dendritic complexity of neocortical Layer II/III CPN has also been observed in postmortem brains of RTT patients (Belichenko et al., 1994; Armstrong et al., 1995), with synaptic circuit abnormalities identified in this population in mouse (Wood et al., 2009). In fact, perturbed dendritic complexity of Layer II/III CPN is observed in multiple neurodevelopmental disorders, including ASD (Egaas et al., 1995; Piven et al., 1997; Mukaetova-Ladinska et al., 2004; Herbert and Kenet, 2007; Frazier and Hardan, 2009; Hardan et al., 2009; Srivastava et al., 2012), attention-deficit/hyperactivity disorder (Hynd et al., 1991; Roessner et al., 2004; Seidman et al., 2005), and schizophrenia (Swayze et al., 1990; Tibbo et al., 1998; Innocenti et al., 2003; Wolf et al., 2008). Further, genetic attenuation of the NF-κB pathway improves the reduced complexity of CPN in *Mecp2*-/y mice (Kishi et al., 2016). We thus focused on this important neuronal population as a window into the broader pathophysiology of RTT.

**Figure 4.**
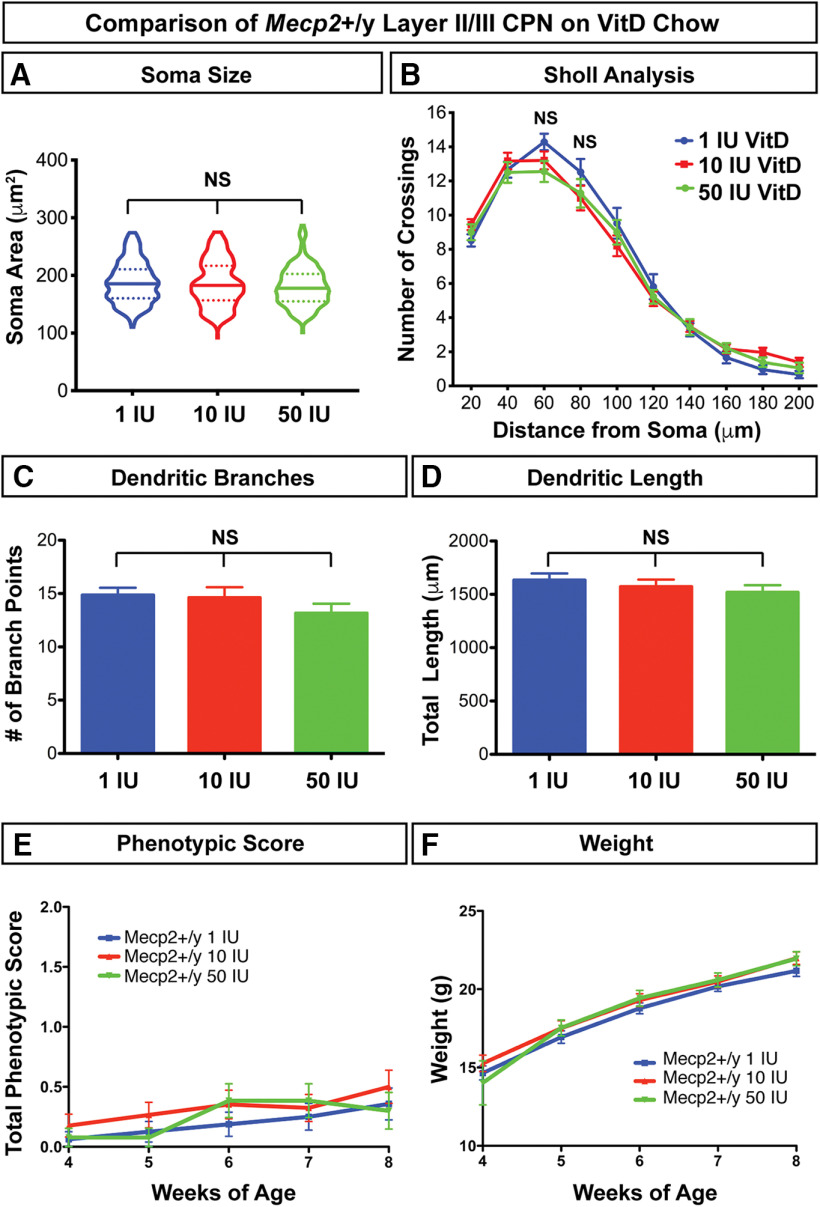
Dietary vitamin D supplementation does not significantly alter neuronal morphology or health in wild-type (*Mecp2*+/y) mice. Treatment of *Mecp2*+/y mice with vitamin D supplemented chow between four and eight weeks of age does not alter (***A***) soma size (*p* = 0.67, one-way ANOVA; 1 IU/g *n* = 76, 10 IU/g *n* = 103, 50 IU/g *n* = 84) or (***B–D***) dendritic complexity of Layer II/III pyramidal neurons, as measured by Golgi staining and (***B***) Sholl analysis, (***C***) quantification of the number of dendritic branches, or (***D***) quantification of total dendritic length (1 IU/g *n* = 20 neurons, 10 IU/g *n* = 29, 50 IU/g *n* = 18). In addition, vitamin D supplementation does not alter the (***E***) total phenotypic score (*p* = 0.34, one-way ANOVA) or (***F***) weight of *Mecp2*+/y mice (*p* = 0.66, one-way ANOVA). ***B***, ***E***, ***F***, Two-way ANOVA with Bonferroni *post hoc* tests. ***A***, ***C***, ***D***, One-way ANOVA with Tukey’s *post hoc* tests. NS = not significant. Error bar: ±SEM.

**Figure 5.**
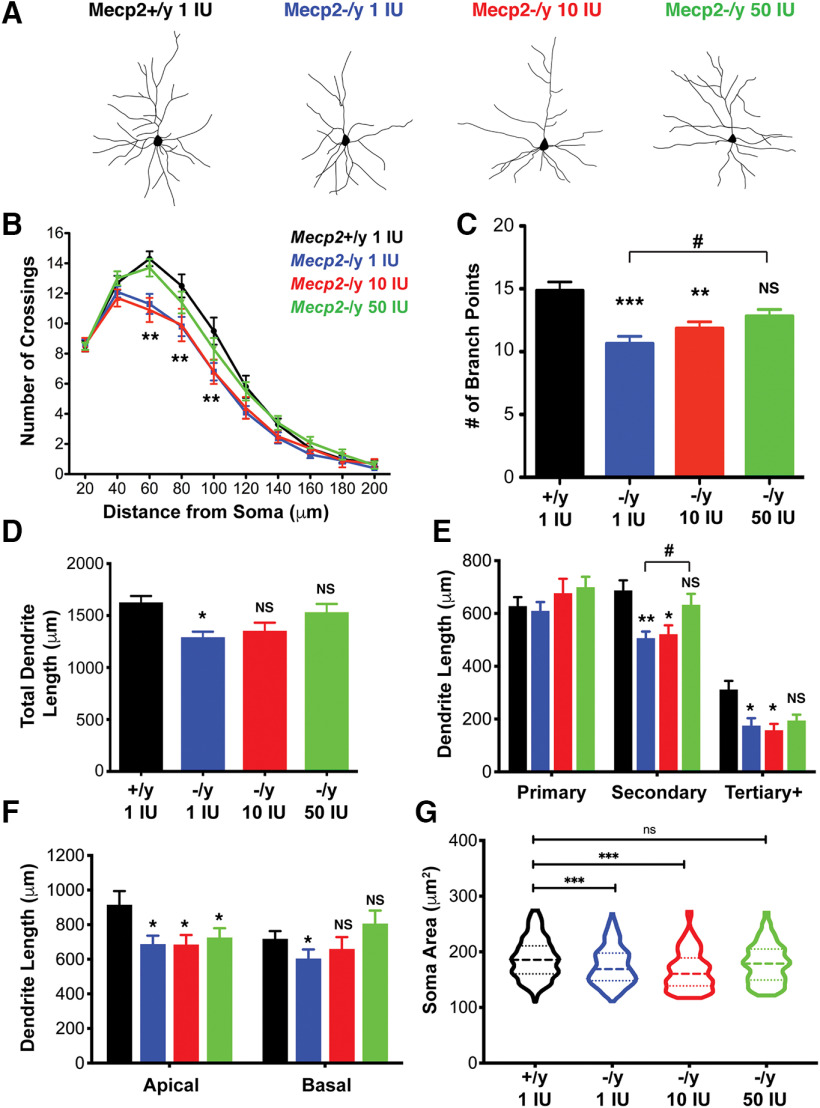
Vitamin D supplementation rescues reduced cortical dendritic complexity and soma size phenotypes in *Mecp2*-null mice. ***A***, Representative traces of Layer II/III cortical CPN following Golgi staining. ***B–F***, Dendritic complexity of CPN, as measured by (***B***) Sholl analysis, (***C***) number of branch points, and (***D***) total dendritic length, is significantly reduced in *Mecp2*−/y mice on both control 1 IU/g and 10 IU/g VitD chow, compared with *Mecp2*+/y on control 1 IU/g chow. Dendritic complexity of *Mecp2*-/y mice on 50 IU/g VitD, however, is essentially indistinguishable from wild type (*Mecp2*+/y). ***E***, *Mecp2*−/y mice on both control 1 IU/g and 10 IU/g VitD chow have reduced secondary and tertiary dendrite lengths, which are rescued in *Mecp2*−/y mice on 50 IU/g VitD. ***F***, The length of apical dendrites is also significantly lower in *Mecp2*-nulls on all chows, compared with wild-type mice. However, the length of basal dendrites of *Mecp2*−/y on 10 IU/g VitD and 50 IU/g VitD chow is rescued, and it is not significantly different from *Mecp2*+/y mice. ***G***, Soma area of Layer II/III CPN is significantly reduced in *Mecp2*−/y cortex on both control chow and 10 IU/g VitD chow, relative to *Mecp2*+/y on control chow, but is rescued with 50 IU/g VitD. ***B***, Two-way ANOVA, Bonferroni *post hoc* test. ***C–F***, One-way ANOVA with Tukey’s multiple comparison test; *p < 0.05, **p < 0.01, ***p < 0.001, NS = not significant. * Compared with *Mecp2*+/y. # Compared with *Mecp2*−/y 1 IU/g VitD. ***B–F***, N: *Mecp2*+/y IU = 21 neurons from three brains, *Mecp2*-/y 1 IU = 28 neurons from four brains, 10 IU = 19 neurons from three brains, 50 IU = 35 neurons from five brains. ***G***, N: *Mecp2*+/y IU = 228 neurons from three brains, *Mecp2*-/y 1 IU = 263 neurons from four brains, 10 IU = 193 neurons from three brains, 50 IU = 204 neurons from five brains. Error bar: ±SEM.

Supplementing with vitamin D between four and eight weeks of age has no significant effect on dendritic complexity or soma size of CPN in wild-type mice (Fig. 4*A–D*), nor does it affect overall health (measured by total phenotypic score) or weight of wild-type mice (Fig. 4*E*,*F*). Thus, for clarity and rigor, we compare *Mecp2*−/y mice on all vitamin D concentrations to wild-type (*Mecp2*+/y) mice on 1 IU/g (control) chow in subsequent analyses. Strikingly, we find that supplementation with 50 IU/g vitamin D fully rescues the reduced dendritic complexity of *Mecp2*-null Layer II/III CPN, as measured by Golgi staining and Sholl analysis (Fig. 5*A*,*B*). This rescue appears to result from both an increase in the number of branch points, relative to *Mecp2*-/y on control chow (Fig. 5*C*), and total dendritic length (Fig. 5*D*).

Further evaluation of the data reveals that the total dendritic length reduction in *Mecp2*-/y CPN is not due to primary dendrites, but, rather, to reduced secondary and tertiary+ dendrites. Strikingly, these secondary and tertiary dendrites in *Mecp2*-/y mice receiving 50 IU/g of vitamin D supplementation are not significantly different from wild-type (Fig. 5*E*). However, this rescue is limited to basal dendrites; the total basal dendritic length of *Mecp2*-/y CPN on vitamin D supplementation is not significantly different from wild type, while the apical dendritic branches continue to show significant reduction in length (Fig. 5*F*). Further, 50 IU/g vitamin D, but not 10 IU/g, rescues the reduced soma size of *Mecp2*−/y Layer II/III CPN (Fig. 5*G*). It is likely that the morphologic abnormalities observed in this neuronal population underlie at least some aspects of the cognitive, behavioral phenotypes observed in RTT, suggesting that amelioration of these phenotypes via vitamin D supplementation might potentially alleviate some RTT symptoms.

### Dietary vitamin D supplementation rescues dendritic spine density of *Mecp2−*/y CPN

**Figure 6.**
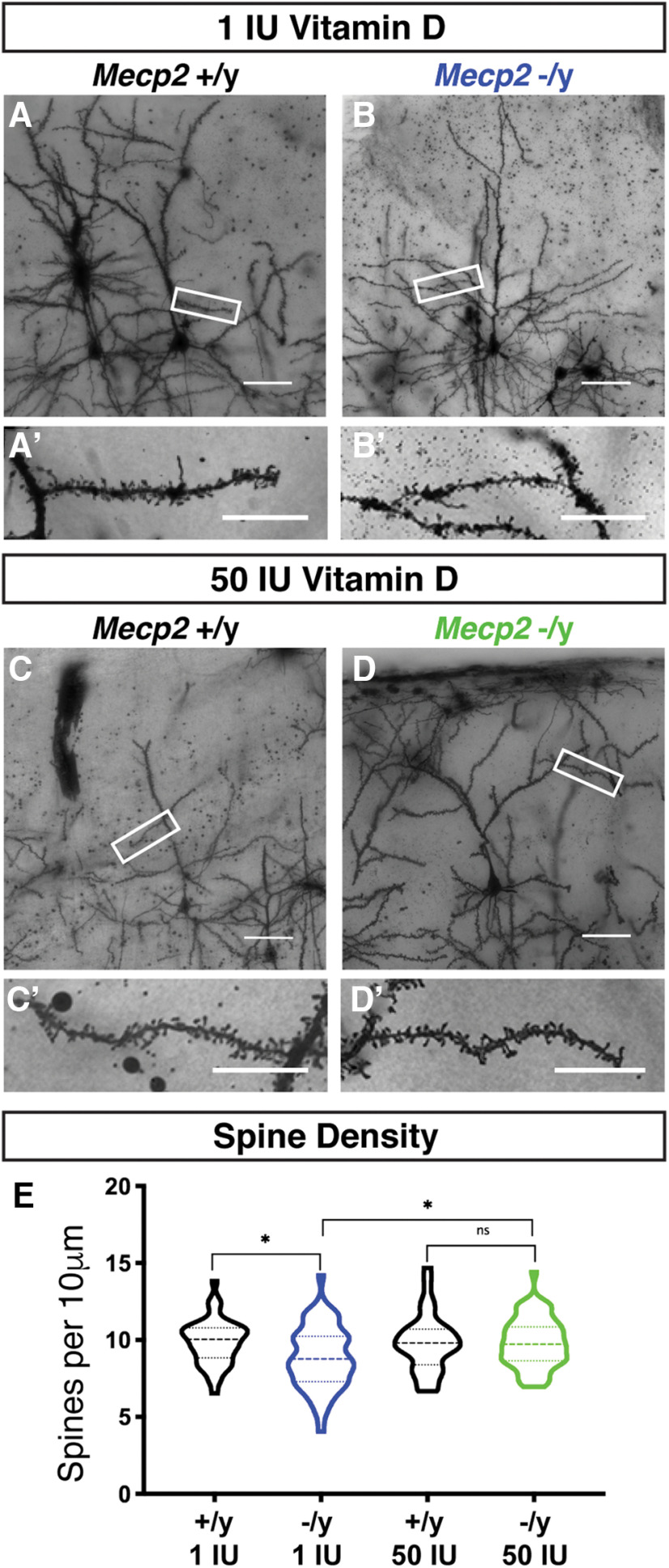
Vitamin D supplementation rescues reduced dendritic spine density in *Mecp2*-/y Layer II/III CPN. ***A–D***, Representative images of apical dendrites of Layer II/III CPN in somatosensory cortex following Golgi staining. Boxes indicate areas displayed at higher magnification in ***A’–D’***. ***E***, Spine density is significantly decreased in *Mecp2*-null neurons compared with wild-type littermates. This decrease is rescued with 50 IU/g vitamin D supplementation; *p < 0.05, one-way ANOVA with Tukey’s multiple comparison test. *N* = *Mecp2*+/y 1 IU: 43 dendrites from three brains, *Mecp2*−/y 1 IU: 54 dendrites from three brains, *Mecp2*+/y 50 IU: 33 dendrites from three brains, *Mecp2*-/y 50 IU: 64 dendrites from four brains. Scale bar = 200 μm (***A–D***) and 5 μm (***A’–D’***). ns = not significant. Error bar ±SEM.

In addition to alterations in dendritic complexity and soma area of cortical neurons, RTT patients and *Mecp2* mutant mice are known to have reduced dendritic spine density (Belichenko et al., 1994, 2009; Armstrong et al., 1995; Fukuda et al., 2005). To investigate whether vitamin D might rescue this phenotype, we analyzed apical dendrites of Layer II/III cortical projection neurons in the neocortex of eight-week-old *Mecp2*-null and wild-type mice on control (1 IU/g) and 50 IU/g vitamin D chow (Fig. 6*A–D*). We focused our analyses on 50 IU/g vitamin D because this concentration rescues both dendritic complexity and soma size of the neurons. The data reveal significant reduction in spine density in the apical dendrites of *Mecp2*−/y mice on control chow, when compared with wild-type littermates. Vitamin D supplementation, however, fully rescues the decreased dendritic spine density of *Mecp2−/y* CPN, while not significantly altering the number of dendritic spines in wild-type littermates (Fig. 6*E*). Together, these results indicate that dietary vitamin D supplementation is able to rescue reduced neuronal size and complexity of *Mecp2*-null neurons but does not modify morphology of wild-type neurons.

### Female *Mecp2* heterozygous mice also display aberrant NF-κB pathway activation

Although RTT is an X-linked disorder, and human males with a mutation in *MECP2* rarely survive past birth, *Mecp2* loss-of-function is less severe in mice. Male hemizygous null mice not only survive until adulthood, they have been the most commonly studied model system. Heterozygous female mice (*Mecp2*+/−) have not been as thoroughly characterized, likely because of the added experimental challenges that they present, including delayed and more variable phenotypic progression, and cellular mosaicism due to X-inactivation (Guy et al., 2001; Samaco et al., 2013; Vogel Ciernia et al., 2017; Ribeiro and MacDonald, 2020). However, they are a more clinically relevant RTT model, and it has become a consensus opinion that it is imperative to include female *Mecp2*+/− for optimal information in studies of potential therapeutics (Katz et al., 2012).

**Figure 7.**
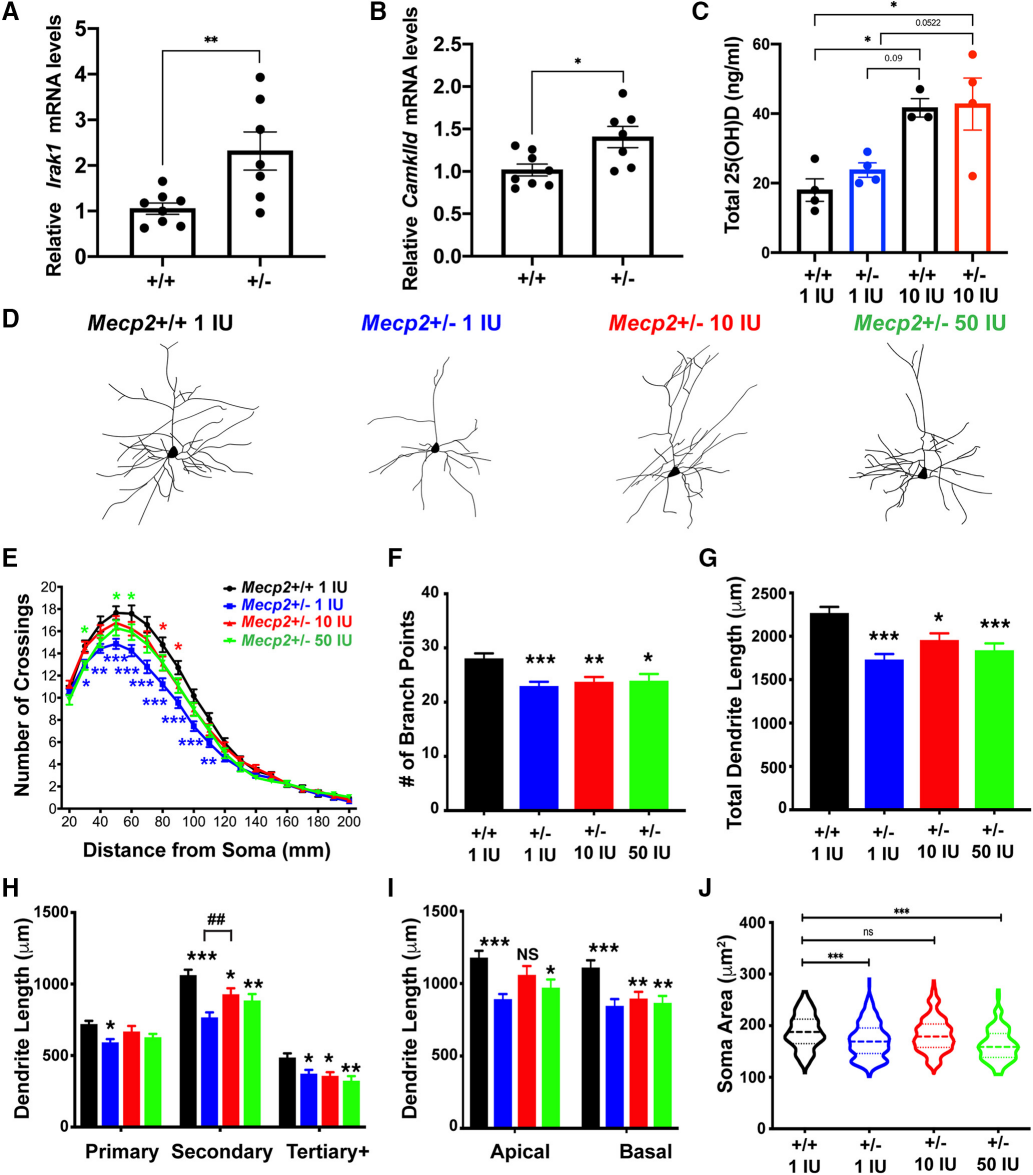
*Mecp2*+/− female cortex has increased *Irak1* expression, and displays partial rescue of reduced dendritic complexity and soma size phenotypes with vitamin D (VitD) supplementation. ***A***, Female *Mecp2*+/− cortex also displays upregulation of *Irak1* expression at five months, as previously determined in male *Mecp2*-/y cortex at eight weeks (two-tailed *t* test, *p* = 0.009; *N*: *Mecp2*+/+ = 8, *Mecp2*+/− = 7). ***B***, Five-month-old *Mecp2*+/− mice show increased expression of the NF-κB downstream target *CamkIId* (two-tailed *t* test, *p* = 0.015; *N*: *Mecp2*+/+ = 8, *Mecp2*+/− = 7). ***C***, *Mecp2*+/− females on control chow (1 IU) do not display lower levels of VitD at five months of age; however, supplementing the diet of the mice with 10 IU/g VitD from four weeks of age significantly increases total serum levels of 25(OH)D, independent of genotype. ***D***, Representative traces of Layer II/III cortical CPNs. ***E***, At five months of age, *Mecp2*+/− mice on both 10 IU/g and 50 IU/g VitD have increased dendritic complexity compared with *Mecp2*+/− on control 1 IU/g chow, as measured by Golgi staining and Sholl analysis, although it is not fully rescued to wild-type (*Mecp2*+/+) levels. Asterisks denote significant difference for *Mecp2*+/− on 1 IU (blue), 10 IU (red), and 50 IU/g VitD (green) compared with *Mecp2*+/+ on control chow. ***F***, ***G***, *Mecp2*+/− on all VitD chows show reduced number of branch points (***F***) and total dendritic length (***G***) compared with wild type, although there is a trend toward increased branch points and dendrite length with VitD supplementation. ***H***, ***I***, *Mecp2*+/− mice on 10 IU/g VitD demonstrate a significant increase in secondary dendrite length relative to control chow (***H***), and apical dendritic length that is not significantly different from wild type (***I***). ***J***, *Mecp2*+/− mice on 10 IU/g VitD chow also show increased soma area, which is not significantly different from *Mecp2*+/+ mice on control chow. ***C***, One-way ANOVA with Tukey’s multiple comparisons test. ***E***, Two-way ANOVA with Bonferroni *post hoc* test. ***F–I***, One-way ANOVA with Tukey’s *post hoc* test. ***C***, *N*: *Mecp2*+/+ 1 IU, *Mecp2*+/− 1 IU and *Mecp2*+/− 10 IU = 4 animals, *Mecp2*+/+ 10 IU = 3 animals. ***E–I***, *N*: *Mecp2*+/+ 1 IU = 46 neurons from five brains, *Mecp2*+/− 1 IU = 68 neurons from six brains, 10 IU = 62 neurons from six brains, 50 IU = 47 neurons from five brains. ***J***, *N* = *Mecp2*+/+ 1 IU = 192 neurons from five brains, *Mecp2*+/− 1 IU = 366 neurons from six brains, 10 IU = 323 neurons from six brains, 50 IU = 234 neurons from five brains; *p < 0.05, **p < 0.01, ***p < 0.001. NS = not significant. Error bar: ±SEM.

We first investigated whether female *Mecp2*+/− also display aberrant NF-κB pathway activation. Overexpression of *Irak1*, encoding a signaling kinase and scaffold protein within the NF-κB pathway, is highly prevalent in male *Mecp2-/y* mice, identified in a transcriptome study from CPN (Kishi et al., 2016), as well as in studies from other brain regions and different strains (Gabel et al., 2015). Overexpression of *Irak1* leads to aberrant NF-κB pathway activation and NF-κB pathway attenuation can rescue the reduced dendritic complexity of *Mecp2*-null neurons and extend the usually shortened lifespan of male *Mecp2*-null mice (Kishi et al., 2016). We find that *Irak1* is significantly upregulated in the cortex of *Mecp2*+/− mice as well (Fig. 7*A*). Additionally, *CamkIId*, a downstream target of the NF-κB pathway, is upregulated in the cortex of *Mecp2*+/− mice when compared with their wild-type littermates (Fig. 7*B*), as previously reported in *Mecp2*-/y animals (Kishi et al., 2016). These results support the conclusion that aberrant NF-κB pathway activation is also prevalent within the female *Mecp2*+/− neocortex and contributes to their neuronal phenotypes.

### Vitamin D supplementation partially rescues reduced CPN dendritic complexity in female heterozygous *Mecp2+/−* mice

To investigate whether *Mecp2*+/− mice also display improvement of neuronal morphology phenotypes with vitamin D supplementation, *Mecp2*+/− and wild-type littermates (*Mecp2*+/+) were placed on custom chow at four weeks of age, as outlined for males. Vitamin D serum levels and dendritic complexity were analyzed at five months, an age at which cortical dendritic complexity and soma size phenotypes are already apparent (Rietveld et al., 2015) and *Mecp2*+/− mice consistently display motor impairments (Samaco et al., 2013). Unlike *Mecp2*-null mice, *Mecp2*+/− females on control chow do not display significantly reduced levels of 25(OH)D under control conditions. However, 10 IU/g vitamin D dietary supplementation significantly increases 25(OH)D serum levels for both *Mecp2*+/+ and *Mecp2*+/− mice (Fig. 7*C*). Supplementation with both 10 and 50 IU/g vitamin D significantly increases Layer II/III CPN dendritic complexity in *Mecp2*+/− cortex, compared with *Mecp2*+/− on control chow, although it does not fully rescue to wild-type complexity (Fig. 7*D*,*E*). Similar to *Mecp2*-/y males, *Mecp2*+/− females exhibit a reduced number of branch points and total dendritic length compared with their wild-type littermates. Although vitamin D supplementation does not fully rescue these phenotypes, there is a trend toward increased total dendritic length with vitamin D supplementation, particularly 10 IU/g vitamin D (Fig. 7*F*,*G*). *Mecp2*+/− mice on 10 IU/g vitamin D demonstrate a significant increase in secondary dendrite length, relative to *Mecp2*+/− on 1 IU/g vitamin D, with *Mecp2*+/− on both 10 and 50 IU/g vitamin D supplemented diets showing primary dendrite length that is not significantly different from wild type (Fig. 7*H*). Intriguingly, *Mecp2*+/− females on 10 IU/g vitamin D chow show a rescue in apical dendritic length (Fig. 7*I*). This differs from the *Mecp2*-null male mice, which demonstrated rescue of the length of their basal dendrites, but not of their apical dendrites. Additionally, supplementation of 10 IU/g vitamin D appears to have the most beneficial effect by also rescuing the reduced soma size of *Mecp2*+/− Layer II/III CPN (Fig. 7*J*).

Together, these results demonstrate that vitamin D supplementation in the 10- to 50-IU/g range ameliorates neuronal size and complexity phenotypes in female heterozygous as well as male hemizygous null mice and further suggests that there might be sex-specific differences in optimal dose, so the treatment paradigm should be optimized independently for each sex. These results have implications more broadly regarding other potential pharmacologic routes to NF-κB inhibition, perhaps contributing to RTT therapy.

## Discussion

In this study, we tested the ability of vitamin D, a simple, cost-effective inhibitor of NF-κB signaling, to rescue the aberrant NF-κB pathway activation in *Mecp2*-mutant neurons and to improve specific RTT phenotypes. We identified a surprisingly efficacious, dose-dependent amelioration of both Layer II/III CPN dendritic complexity and soma size phenotypes, in addition to moderate improvements to overall health and longevity. Our multistage experiments show efficacy in both female *Mecp2*+/− mice that most closely model the human disease, and in male *Mecp2*−/y mice, which have been more widely used in earlier analyses due to their rapid progression. Our results have broader relevance for the potential of NF-κB pathway inhibition to contribute to therapeutic approaches for RTT, with a range of increasingly specific, controllable, and potentially targetable inhibitors of this pathway in existence or under development. That said, vitamin D provides more than simply a proof-of-concept, since it is already known to be safe, has no or little toxicity at the dosage ranges in question, and also directly addresses known vitamin D deficiency in RTT patients.

The NF-κB pathway regulates many cellular processes, including immune response, and *Mecp2* knock-down has also been found to lead to enhanced NF-κB signaling in myeloid lineage cells (O'Driscoll et al., 2013, 2015). NF-κB subunits are also expressed throughout the CNS, and there is an extensive literature implicating the NF-κB pathway in regulation of neural process development and structural plasticity, in addition to learning and memory (Gutierrez and Davies, 2011). Further, previous results demonstrate that genetic attenuation of this pathway in *Mecp2*-/y mice rescues RTT phenotypes (Kishi et al., 2016), and it has been shown that inhibition of the Gsk3b pathway improves neuronal morphology in *Mecp2*-null neurons by reducing NF-κB signaling (Jorge-Torres et al., 2018). Together, these results indicate that abnormal activation of NF-κB signaling contributes to the pathogenesis of *Mecp2*-null mice, and likely RTT. The broad neurologic phenotypes of RTT overlap with multiple other neurologic disorders, both neurodevelopmental (e.g., ASD, some forms of cerebral palsy, and epilepsy) and acquired (e.g., traumatic brain injury), raising interesting questions regarding converging underlying mechanisms and possible involvement of NF-κB signaling, either causal or potentially permissive for enhanced recovery. Thus, NF-κB pathway inhibition might provide a novel therapeutic target not only for the devasting disorder RTT but also potentially to treat elements of neurologic disorders with overlapping pathology.

Previous studies have identified other compounds, such as rhIGF1, ketamine, and cannabidivarin, that appear to also significantly improve behavioral and morphologic phenotypes of *Mecp2* mutant mice (Castro et al., 2014; Patrizi et al., 2016; Zamberletti et al., 2019). Further, genetic attenuation of the NF-κB pathway (Kishi et al., 2016) is more effective at rescuing *Mecp2*-null lifespan than our early-symptomatic vitamin D supplementation (Fig. 3), suggesting that either earlier onset of NF-κB pathway inhibition and/or other, more specific NF-κB inhibitors might be more efficacious. That said, our results reported here offer a straightforward, readily implementable, and immediately available option: vitamin D, which is cost-effective and of easy access. For this reason, our work strongly motivates that vitamin D supplementation be more thoroughly investigated as a simple, partial therapeutic avenue for RTT, likely in combination with other approaches.

Although the vitamin D deficiency repeatedly observed in RTT patients has been largely attributed to poor nutrition and/or lack of exposure to sunlight, our results that *Mecp2*-null mice that are maintained in a controlled environment on chow considered to be vitamin D sufficient also have reduced vitamin D serum levels (Fig. 1) suggest an underlying deficiency. One potential mechanism contributing to this vitamin D deficiency could be the disrupted cholesterol homeostasis reported in *Mecp2*-null mice (Buchovecky et al., 2013), since the primary natural source of vitamin D is dermal synthesis from cholesterol. The findings that heterozygous female mice maintained in the controlled environment do not display reduced vitamin D serum levels might indicate that their ∼50% mosaic of MeCP2+ cells is sufficient to maintain the synthesis of vitamin D. However, increased vitamin D still partially rescues the neuronal morphology phenotypes. Thus, it is interesting to speculate that *Mecp2*+/− maintained on a vitamin D deficient diet might likely have more severe phenotypes, perhaps more closely resembling the male *Mecp2*−/y mice.

In addition to the vitamin D receptor (VDR), which is mainly expressed in the nucleus of cells within the brain, protein disulfide isomerase family member 3 (PDIA3) is a known VDR localized in the cellular membrane (Nemere et al., 2004; Eyles et al., 2014). PDIA3 is associated with rapid nongenomic response to vitamin D, although both receptors are thought to work in conjunction (Boyan et al., 2012; Chen et al., 2013a). While the expression of *Vdr* is very low in the brain compared with kidney and liver of rodents, *Pdia3* displays greater abundance in brain than in other organs (Landel et al., 2018). We found no difference in *Pdia3* expression in the cortex of either *Mecp2* mutant male (*p* = 0.56) or female mice (*p* = 0.84), suggesting that they do not have a disruption in their ability to respond to vitamin D.

Our data suggest that vitamin D can act directly on cortical neurons to rescue their reduced dendritic complexity *in vitro*, with complementary work by us and by others indicating primarily direct action with regard to dendritic complexity. *Mecp2* mutant cortical phenotypes result from both cell-autonomous and cell non-autonomous disruptions (Ribeiro and MacDonald, 2020). For example, reciprocal cross-transplantation studies demonstrate that *Mecp2*-/y CPN display reduced dendritic complexity even in the context of a wild-type cortex, but that soma size is dependent on the recipient cortical *Mecp2* genotype (Kishi and Macklis, 2010). Further, in heterozygous females, dendritic complexity of Layer V cortical neurons correlates with MeCP2 cell-autonomous expression, while soma size is reduced even in wild-type neurons (Rietveld et al., 2015). In addition, the molecular pathways regulated by MeCP2 are tissue and cell-type specific (Samaco et al., 2009; Chao et al., 2010; Lioy et al., 2011; Derecki et al., 2012; Sugino et al., 2014), and loss of MeCP2 function in defined CNS circuits results in distinct RTT phenotypes (Alvarez-Saavedra et al., 2007; Fyffe et al., 2008; Adachi et al., 2009; Ward et al., 2011; Nguyen et al., 2013; Wither et al., 2013; He et al., 2014). NF-κB signaling is prevalent in glia, and the VDR is expressed by both neurons and astrocytes (Eyles et al., 2005). Thus, vitamin D might act on distinct cellular targets to differentially improve specific RTT phenotypes.

Interestingly, we also observe a sex difference in how *Mecp2* mutant mice respond to our supplementation paradigm, though both sexes display increased circulating 25(OH)D after vitamin D dietary supplementation. Male *Mecp2*-null mice demonstrate CPN morphologic rescue when treated with 50 IU/g of vitamin D (Figs. 5, 6) while heterozygous females respond better to 10 IU/g of vitamin D supplementation (Fig. 7). Furthermore, vitamin D supplementation rescues basal dendrite length in *Mecp2*-/y cortex (Fig. 5), and apical dendrite length in *Mecp2*+/− females (Fig. 7). Different genes selectively control basal or apical dendritic maintenance (de Anda et al., 2012; Srivastava et al., 2012; Pathania et al., 2014; Cubelos et al., 2015; Rietveld et al., 2015). Therefore, it is tempting to speculate that the distinct treatment responses we see in males and females might be a result of different genes responding to *Mecp2* mosaic expression in *Mecp2*+/− mice and/or to non-cell autonomous effects regulating dendritic branching. Another consideration is the duration of the treatment: while mice of both sexes were placed on custom chow when weaned at P28, *Mecp2*-null male mice treatment lasted only four weeks, due to their shortened lifespan, while heterozygous female mice were on the custom diet for four months until reaching a typical symptomatic age.

In summary, we identify that dietary vitamin D supplementation, within a widely acceptable and nontoxic dosage range, rescues aberrant NF-κB pathway activation and partially ameliorates downstream neuropathological effects of NF-κB signaling in *Mecp2* mutant mice. These results further solidify the NF-κB pathway as a potential novel therapeutic target for RTT. We demonstrate that vitamin D inhibits this pathway in *Mecp2* knock-down neurons *in vitro*, ameliorates reduced neocortical dendritic morphology and soma size phenotypes in a dose-dependent manner *in vivo* in both male and female RTT model mice, and modestly improves the reduced lifespan of male *Mecp2*-null mice. While it is known that neuronal morphologic rescue can lead to behavioral improvements of *Mecp2*-null mice (Bu et al., 2017; Chin et al., 2019), it will be important for future studies to assess both complex mouse behavior and electrophysiological properties of *Mecp2*-null neurons in mice with vitamin D supplementation, to further investigate the breadth of therapeutic potential of vitamin D supplementation and the specific phenotypes that are or are not improved. Together, our results both provide new insight into the fundamental neurobiology of RTT, and motivate consideration of NF-κB pathway inhibition, including via vitamin D dietary supplementation, as a potential partial therapeutic intervention for RTT.

## Synthesis

Reviewing Editor: Margaret McCarthy, Univ of Maryland School of Medicine Decisions are customarily a result of the Reviewing Editor and the peer reviewers coming together and discussing their recommendations until a consensus is reached. When revisions are invited, a fact-based synthesis statement explaining their decision and outlining what is needed to prepare a revision will be listed below. The following reviewer(s) agreed to reveal their identity: NONE. Note: If this manuscript was transferred from JNeurosci and a decision was made to accept the manuscript without peer review, a brief statement to this effect will instead be what is listed below.

The authors have done an excellent job of responding to the previous reviewers. This is a very nice manuscript, the figures are a model for how to communicate clearly.
